# Specific dairy foods and risk of frailty in older women: a prospective cohort study

**DOI:** 10.1186/s12916-024-03280-8

**Published:** 2024-02-29

**Authors:** Ellen A. Struijk, Teresa T. Fung, Fernando Rodriguez-Artalejo, Heike A. Bischoff-Ferrari, Walter C. Willett, Esther Lopez-Garcia

**Affiliations:** 1 Department of Preventive Medicine and Public Health, School of Medicine, Universidad Autónoma de Madrid-IdiPaz, Avda, Arzobispo Morcillo, 4, 28029 Madrid, Spain; 2 CIBERESP (CIBER of Epidemiology and Public Health), Madrid, Spain; 3 Department of Nutrition, Simmons University, Boston, MA USA; 4 Department of Nutrition, Harvard T.H. Chan School of Public Health, Boston, MA USA; 5 IMDEA/Food Institute. CEI UAM+CSIC, Madrid, Spain; 6 Department of Geriatrics and Aging Research, University Hospital Zurich and University of Zurich, Zurich, Switzerland; 7 Centre On Aging and Mobility, University Hospital Zurich and Waid City Hospital, Zurich, Switzerland; 8 Department of Epidemiology, Harvard T.H. Chan School of Public Health, Boston, MA USA

**Keywords:** Dairy, Frailty, Milk, Physical function, Older adults

## Abstract

**Background:**

Dairy contains a complex mixture of lipids, proteins, and micronutrients. Whether habitual dairy consumption is associated with health benefits is not well established. Since dairy is high in nutrients that are potentially protective against frailty, the association between dairy products and the risk of frailty is of interest.

**Methods:**

We analyzed data from 85,280 women aged ≥ 60 years participating in the Nurses’ Health Study. Consumption of milk, yogurt, and cheese was obtained from repeated food frequency questionnaires administered between 1980 and 2010. Frailty was defined as having at least three of the following five criteria from the FRAIL scale: fatigue, low strength, reduced aerobic capacity, having ≥ 5 chronic illnesses, and a weight loss of ≥ 5%. The occurrence of frailty was assessed every four years from 1992 to 2018. Cox proportional hazard models were used to examine the association between the intake of dairy foods and frailty.

**Results:**

During follow-up we identified 15,912 incident cases of frailty. Consumption of milk or yogurt was not associated with the risk of frailty after adjustment for lifestyle factors, medication use, and overall diet quality. Cheese consumption was positively associated with risk of frailty [relative risk (95% confidence interval) for one serving/day increment in consumption: 1.10 (1.05, 1.16)]. Replacing one serving/day of milk, yogurt, or cheese with one serving/day of whole grains, nuts, or legumes was associated with a significant lower risk of frailty, while replacing milk, yogurt, or cheese with red meat or eggs was associated with an increased risk. When milk was replaced with a sugar-sweetened or artificially sweetened beverage, a greater risk of frailty was observed, while replacing milk with orange juice was associated with a lower risk of frailty.

**Conclusions:**

The results suggest that the association between milk, yogurt, and cheese and frailty partly depends on the replacement product. Habitual consumption of milk or yogurt was not associated with risk of frailty, whereas cheese consumption may be associated with an increased risk.

**Supplementary Information:**

The online version contains supplementary material available at 10.1186/s12916-024-03280-8.

## Background

Frailty is a syndrome characterized by decreased biological reserve and resistance to stressors, causing increased risk of adverse outcomes including falls, disability, hospitalization, and death [1]. The aging of society and the subsequent high and rising prevalence of frailty make prevention and early intervention crucial to ensure healthy aging [2]. A substantial body of evidence suggests that better overall diet quality reduces risk of frailty [3, 4]. Yet, knowledge on the relation of specific dietary factors, such as dairy products, to frailty is still limited.

Dairy products are a nutrient-dense source of protein. Protein is thought to play an important role in the prevention of frailty because deficient intake adversely affects muscle mass and strength [5–7], a disorder known as sarcopenia [8], which is related to the frailty syndrome. However, previous research with the current population has shown that women with a higher intake of dairy protein did not have a lower risk of frailty [9]. Dairy provides several other nutrients potentially protective against frailty, including calcium, magnesium, and vitamin D in whole and fortified dairy products, which may delay sarcopenia through its role in muscle contraction and metabolism [10]. Additionally, dairy intake could contribute to meeting the calcium requirements to slow the degree of bone loss, which in turn may affect the rate frailty advances [11], although the association between calcium and bone density is not well established [12]. On the other hand, many dairy foods add a substantial amount of saturated fat to the diet, which has adverse effects on blood lipids and is positively associated with risk of cardiovascular disease when compared to unsaturated fats [12].

Therefore, we aimed to further explore dairy products. Since milk, yogurt, and cheese vary greatly in nutrient content, structure, processing methods and bioactive ingredients, its association with frailty may differ. The dairy matrix can also influence nutrient interactions, absorption, and digestion [13, 14]. Given that the effect of dairy consumption on frailty is still unclear, we investigated whether dairy foods are associated with this outcome among a large population of older women from the US.

## Methods

### Study design and participants

The Nurses’ Health Study (NHS) was established in 1976 with the enrollment of 121,700 female nurses aged 30 to 55 years at inception [15]. Participants completed biennial mailed questionnaires to update information on medical history and lifestyle. The follow-up rate was approximately 90% at each follow-up cycle. The Harvard T.H. Chan School of Public Health and the Brigham and Women’s Hospital Human Subjects Committee Review Board approved the protocol for the study, and participants provided written informed consent.

For this analysis we included women aged ≥ 60 years at baseline with complete information on the exposure and outcome variables. Women younger than 60 years entered the study when they turned 60 during subsequent questionnaire cycles. Women with an implausible high (> 3500 kcal/d) or low (< 500 kcal/d) energy intake were excluded, as well as women identified as frail at the analytical baseline, leaving a total population of 85,280 women for analysis. The association between dairy consumption and frailty occurrence was examined up to 2018.

### Dietary assessment

Dietary intake was assessed using a validated food frequency questionnaire (FFQ) in 1980, 1984, 1986, 1990, 1994, 1998, 2000, 2006 and 2010, as described in detail elsewhere [16]. In each questionnaire, participants were asked how often on average during the previous year they consumed the foods specified. A standard portion size based on nutritionists´ experience and nine possible responses for the frequency of consumption (¨never, or less than once per month¨; ¨1–3 per month¨; ¨once per week¨; ¨2–4 per week¨; ¨5–6 per week¨; ¨once per day¨; ¨2–3 per day¨; ¨4–5 per day¨; ¨6 or more times per day¨) [17], were given for each food item. Nutrient and energy intakes were calculated by multiplying the consumption of each food recorded by its nutrient and energy content, using the US Department of Agriculture database, and complemented with information from the manufacturers, and summed across all foods.

Questionnaire items on milk included skim, 1–2% fat, and whole milk and was reported per 1 cup (8 oz) serving. Yogurt was assessed as plain, regularly sweetened, or artificially sweetened and was also reported per 1 cup (8 oz) serving. Cheese included cottage or ricotta cheese (4 oz), cream cheese (1 oz), and other cheeses (e.g., American, cheddar; 1 slice or 1 oz). Regarding the category of other cheeses, participants were asked whether they usually ate regular or low-fat/non-fat cheese. Other dairy foods included in the FFQ were regular ice cream per ½ cup, frozen yogurt or low-fat ice cream per ½ cup, and cream or sour cream per 1 tablespoon (15 mL). The reproducibility and validity of these FFQs have been reported in detail elsewhere [18, 19]. Briefly, the Pearson correlation coefficients between FFQs and multiple diet records ranged between 0.57 (hard cheese) and 0.97 (yogurt) for dairy products [17]. To best represent long-term diet during follow-up and to account for changes in food consumption, we used the cumulative average of dairy consumption from all available dietary questionnaires from 1980 through frailty onset or the end of follow-up [20]. For example, the average dairy intake of 1980, 1984, 1986 and 1990 was used to predict frailty occurrence from 1992 to 1996, and the average of 1980, 1984, 1986, 1990 and 1994 intake was used to predict risk from 1996 to 2000, and so on. When dietary information at follow-up was unavailable the value from the previous cycle was carried forward.

The modified Alternate Healthy Eating Index (AHEI) score was calculated as an indicator of overall diet quality. This score is based on 10 food products and nutrients, including fruit, vegetables, nuts, legumes, whole grains, long chain omega-3 and other polyunsaturated fats, alcohol, red and processed meat, sodium, trans fat, and sugar-sweetened beverages [21]. A higher score in the AHEI denotes better diet quality.

### Frailty assessment

We used the FRAIL scale [22] that includes five self-reported frailty criteria: fatigue, low strength, reduced aerobic capacity, having several chronic illnesses, and significant unintentional weight loss. In 1992, 1996, 2000, 2004, 2008, 2012 and 2016 participants completed the Medical Outcomes Study Short-Form (SF-36), a 36-item-questionnaire with eight health dimensions, including physical and mental components [23]. From the SF-36, we assessed the first three frailty criteria with the following questions: a) for fatigue: “Did you have a lot of energy?”, with response “a little of the time” or “none of the time” (in 1992, 1996 and 2000), or with the question “I could not get going” (in 2004), with response “moderate amount” or “all of the time”, or with the question “I feel full of energy” (in 2008, 2012 and 2016), with response “no”; b) for low strength: “In a normal day, is your health a limitation to walk up 1 flight of stairs?”, with response “yes, limited a lot”; and c) for reduced aerobic capacity: “In a normal day, is your health a limitation to walk several blocks or several miles?”, with response “yes, a lot”. In addition, the illness criterion was assessed from the question “In the last 2 years, have you had any of these physician-diagnosed illnesses?”. We considered that this criterion was met when participants reported ≥ 5 of the following diseases: cancer, hypertension, type 2 diabetes, angina, myocardial infarction, stroke, congestive heart failure, asthma, chronic obstructive lung disease, arthritis, Parkinson’s disease, kidney disease, and depression. Finally, because weight of the participants was available only biannually, we considered the weight loss criterion as a 5% decrease in the weight reported in a 2-year period before the assessment of frailty. At the end of each 4-year follow-up cycle incident frailty was defined as having ≥ 3 criteria in the scale. Missing response in 3 or more components was assumed as missing on frailty status and excluded. For those with one or two missing responses, we were able to assess frailty status considering missing in each characteristic as not having it. The FRAIL scale has been correlated (*r* = 0.62, *p* < 0.001) with the physical frailty phenotype [24], the most widely used scale for frailty assessment, which includes both self-reported (exhaustion; low physical activity) and performance-based measures (grip strength; walking speed; and unintentional weight loss).

### Ascertainment of mortality

Deaths were reported by next of kin, or the postal system, or ascertained through the National Death Index. Follow-up for mortality was more than 98% complete [25]. We obtained copies of death certificates and medical records to determine causes of death (classified according to the categories of the International Classification of Diseases, Ninth Revision). Death records were reviewed and coded by physicians.

### Socioeconomic variables, medical history, anthropometric data, and lifestyle factors

In the analytic baseline questionnaire (1990), we collected information on age, indicators of socioeconomic status (education level, census track income), weight, smoking status, and medication use. This information has been updated on each of the subsequent biennial questionnaires. To calculate body mass index (BMI), we used information on height reported in 1976, when the cohort was initiated and self-reported weight; BMI was calculated as weight in kilograms divided by the square of height in meters. Discretionary physical activity was reported as the average time spent per week during the preceding year in specific activities (e.g., walking outdoors, jogging, and bicycling). The time spent in each activity was multiplied by its typical energy expenditure, expressed in metabolic equivalent tasks, and then summed over all activities. Detailed information on the validity and reproducibility of self-reported weight and physical activity has been published elsewhere [26, 27].

### Statistical analysis

Participants were classified into categories of usual consumption of milk, yogurt, and cheese. We used cause-specific proportional hazards models to calculate relative risks (RR), estimated by hazard ratios, and their 95% confidence interval (CI) for the studied associations, adjusting for potential confounders updated at each four-year cycle. Person-years were calculated from baseline until the occurrence of frailty, death, or the end of the study period (1 June 2018), whichever came first. We stratified the analysis jointly by age in months at start of follow-up and calendar year of each questionnaire cycle.

Multivariable models were adjusted for census tract income (< $45,000, $45,000–$59,999, $60,000–$74,999, $75,000–$99,999, or ≥ $100,000/y), education (registered nursing degrees, bachelor’s degree, masters or doctorate degree), BMI (< 25.0, 25.0–29.9, ≥ 30.0 kg/m^2^), smoking status (never, past, and current 1–14, 15–24, and ≥ 25 cigarettes/day), alcohol intake (0, 1.0–4.9, 5.0–14.9, ≥ 15.0 g/d), energy intake (quintiles of kcal/d), and medication use (yes/no) including postmenopausal hormone therapy, aspirin, diuretics, beta blockers, calcium channel blockers, angiotensin converting enzyme inhibitors, other antihypertensive medication, lipid lowering medication, insulin, and oral hypoglycemic medication. Medication use was included in the model to address the fact that persons with risk factors for chronic diseases are possibly at greater risk of developing frailty, although some over adjustment might exist. Similarly, because the inclusion of BMI might also represent some over adjustment, since weight loss is part of the frailty outcome, BMI was not updated and only BMI measured at baseline was included in the analysis. Results were further adjusted for the overall diet by including the AHEI score as an indicator of diet quality (quartiles). This model additionally included mutual adjustment for each type of dairy (categories). Since physical activity is closely related to the outcome, adjustment for this variable was only done in secondary analyses. A missing indicator variable was created for each covariate with missing values. Tests for linear trends were conducted by modeling intake as a continuous variable.

With substitution analysis, we estimated the effect of replacing one serving/d of milk, yogurt, or cheese consumption with one serving/d of another source of protein (including soy, nuts, legumes, whole grains, or red meat, fish, and eggs) on frailty risk. In another substitution analysis the effect of replacing one serving/d of milk with one serving/d of another beverage (including coffee, tea, water, sugar-sweetened beverage, artificially sweetened beverage, orange juice, or other fruit juice) was examined. Of note, correlations between dairy and the substitution foods were low, with the highest correlation coefficient for milk and whole grains, with a value of 0.28. To fit these models, we simultaneously included all food products we aimed to compare but omitting the dairy product of interest, together with a variable for the total consumption of all replacement food products of interest to control for the total intake, along with the covariates listed above. Since several protein sources overlap with the AHEI score this variable was not included, however in additional analysis the substitution of protein sources was additionally adjusted for sugar sweetened beverages (quintiles), and the substitution of drinks for fruits and vegetables (quintiles) as a marker of a healthy diet.

In addition, several sensitivity analyses were performed. To assess the association between dairy and risk of frailty independent of the protein intake we additionally adjusted the main analysis and substitution analysis for total protein intake. We associated subgroups of the dairy categories depending on its fat or sugar content, including low-fat milk, whole milk, plain yogurt, sweetened yogurt, low-fat cheese, and high-fat cheese with frailty. The association between milk, yogurt, and cheese consumption was also assessed excluding participants with missing values for any criterion of the FRAIL scale. Additionally, the association between each dairy product and each criterion of the FRAIL scale was assessed. Stratification by BMI level (< 25.0/25.0–29.9/ ≥ 30.0 kg/m^2^) was performed to assess the robustness of the results. We replicated the main analyses among those with 0 (robust) or 1–2 (prefrail) of the frailty criteria at baseline to understand whether the effect of dairy products on frailty may differ depending on the baseline status. Additionally, analyses were repeated excluding women with diabetes, cardiovascular disease, or cancer at baseline or those who developed these diseases during the follow-up to assess the independence of this association from main chronic diseases. Finally, we examined the latency effect of dairy intake using multiple dietary assessments. For example, for a latency period of 6–10 years, we used the 1990 intake for cases diagnosed from 1996 to 2000, the 1994 intake for cases diagnosed from 2000 to 2004, and so on. For a latency of 10–14 years, we used the 1990 intake for cases diagnosed from 2000–2004, and so on. The 0–6-year latency analysis corresponds to the analysis using the most recent dietary intake. All statistical tests were 2-sided with a *p* value < 0.05 and performed using SAS software, version 9.4 for UNIX (SAS Institute Inc, Cary, NC).

## Results

The average (standard deviation) dairy consumption among the participants of the study was 0.83 (0.69) servings/day of milk, 0.13 (0.17) servings/day of yogurt and 0.56 (0.37) servings/day of cheese. Table 1 shows the age-standardized baseline characteristics of the study participants by categories of milk, yogurt, and cheese consumption. Trends over categories of milk intake were not clear, however, compared to women who consumed less than 1 glass of milk a week, those who consumed ≥ 2 glasses of milk a day had a higher physical activity level, were less often current smokers and had a lower alcohol intake. Women with a yogurt consumption of ≥ 5 servings a week had a lower BMI, higher physical activity level, were less often current smokers, had a lower alcohol intake and a higher diet quality, compared to those who never consume yogurt. Those who consume at least 1 serving of cheese a day had a higher BMI, but also a higher physical activity level, and a higher energy and alcohol intake compared to those who consume cheese less than once a week.

**Table 1:** Baseline characteristics according to according to the lowest, middle and highest categories of dairy consumption among women aged ≥ 60y in the Nurses’ Health Study

	**Milk**			**Yogurt**			**Cheese**		
	** < 1/wk**	**2 to 4 /wk**	** ≥ 2/d**	**Never**	**1/wk**	** ≥ 5/wk**	** < 1/wk**	**2 to 4 /wk**	** ≥ 1/d**
Participants, n	11,584	24,304	8007	24,495	15,387	1253	4922	43,517	10,223
Mean age, y	62.5 (2.3)	62.4 (2.2)	63.1 (2.5)	63.0 (2.5)	62.3 (2.1)	62.6 (2.3)	62.8 (2.4)	62.5 (2.2)	62.9 (2.5)
BMI, kg/m^2^	25.2 (4.8)	25.7 (4.7)	25.7 (4.8)	25.6 (4.8)	25.7 (4.6)	24.8 (4.5)	25.1 (4.6)	25.6 (4.7)	26.1 (5.0)
Discretionary physical activity, METs-h/wk	17.7 (23.4)	18.4 (22.9)	20.4 (23.7)	16.7 (21.5)	20.1 (22.7)	26.8 (30.3)	16.8 (23.8)	18.5 (22.3)	21.1 (24.9)
Current smoker, %	17	12	12	20	8	7	15	12	12
Education graduate school, %	2	3	3	2	3	4	2	3	3
Census tract income above 100,000/y, %	22	23	21	18	26	25	21	23	22
Medication use^a^									
Aspirin, %	41	46	46	43	47	44	40	46	46
Postmenopausal hormone therapy, %	37	38	37	34	39	39	35	38	38
Diuretics, %	10	10	9	9	11	6	8	10	11
β-Blockers, %	12	13	13	12	14	11	13	13	12
Calcium channel blockers, %	9	10	10	10	9	7	9	10	10
ACE inhibitors, %	9	10	10	9	10	9	9	10	10
Other blood pressure medication, %	8	9	9	9	9	7	9	9	9
Lipid lowering medication, %, %	13	16	14	13	17	11	18	17	12
Insulin, %	1	2	2	2	1	2	2	2	2
Oral hypoglycemic drugs, %	2	3	3	3	3	2	3	3	3
Dietary intake									
Milk, servings/d	0.05 (0.04)	0.48 (0.12)	2.50 (0.54)	0.77 (0.77)	0.91 (0.71)	1.02 (0.84)	0.73 (0.78)	0.85 (0.72)	0.89 (0.76)
Low-fat milk, servings/d	0.03 (0.04)	0.39 (0.18)	2.15 (0.76)	0.59 (0.71)	0.80 (0.68)	0.89 (0.79)	0.59 (0.71)	0.72 (0.68)	0.76 (0.72)
Whole milk, servings/d	0.01 (0.03)	0.09 (0.15)	0.35 (0.68)	0.18 (0.39)	0.11 (0.25)	0.13 (0.36)	0.14 (0.34)	0.13 (0.30)	0.14 (0.31)
Yogurt, servings/d	0.09 (0.17)	0.12 (0.17)	0.14 (0.21)	0.00 (0.00)	0.20 (0.04)	0.96 (0.34)	0.07 (0.16)	0.12 (0.17)	0.16 (0.22)
Plain yogurt, servings/d	0.07 (0.20)	0.09 (0.22)	0.11 (0.26)	0.00 (0.00)	0.15 (0.21)	0.60 (0.76)	0.06 (0.20)	0.10 (0.21)	0.13 (0.26)
Sweetened yogurt, servings/d	0.04 (0.14)	0.06 (0.14)	0.06 (0.16)	0.00 (0.00)	0.09 (0.16)	0.31 (0.54)	0.04 (0.16)	0.06 (0.15)	0.07 (0.17)
Cheese, servings/d	0.55 (0.45)	0.58 (0.38)	0.61 (0.42)	0.54 (0.43)	0.63 (0.38)	0.75 (0.51)	0.08 (0.04)	0.48 (0.12)	1.37 (0.47)
Low-fat cheese, servings/d	0.13 (0.18)	0.16 (0.18)	0.20 (0.23)	0.13 (0.19)	0.20 (0.18)	0.30 (0.32)	0.02 (0.02)	0.14 (0.11)	0.38 (0.35)
High-fat cheese, servings/d	0.43 (0.39)	0.42 (0.32)	0.41 (0.33)	0.41 (0.36)	0.42 (0.31)	0.44 (0.37)	0.06 (0.03)	0.35 (0.13)	0.99 (0.50)
Energy intake, kcal/d	1520 (424)	1651 (407)	1978 (413)	1655 (445)	1756 (419)	1958 (457)	1445 (416)	1682 (398)	1983 (439)
Alcohol intake, g/d	7.9 (11.4)	6.2 (8.9)	4.5 (7.8)	6.7 (10.6)	5.5 (7.7)	5.3 (7.4)	4.2 (8.4)	5.9 (8.8)	7.6 (10.21)
AHEI score	51.8 (10.0)	52.6 (9.5)	50.9 (9.4)	48.8 (9.4)	54.4 (9.0)	58.1 (9.4)	51.5 (10.4)	52.2 (9.4)	52.6 (9.5)
Number of frailty criteria, %									
0	72	71	71	72	70	77	70	71	71
1	22	23	23	22	23	18	23	23	23
2	6	6	6	6	6	5	6	7	6

*BMI* body mass index, *METs* metabolic equivalent tasks, *ACE* angiotensin converting enzyme, *AHEI* Alternate Healthy Eating Index. Values are means (SD) unless otherwise indicated. Data, except age, were directly standardized to the age distribution of the entire cohort

^a^1 or more times per week

During 26 years of follow-up (median follow-up 16 years), we identified a total of 15,912 incident frailty cases (Table 2). Women with a higher consumption of milk had a slightly higher risk of frailty when analyzed per serving/day increase [relative risk (95% confidence interval): 1.02 (1.00, 1.05)]. This association was attenuated when categories of daily consumption were used and models were adjusted for lifestyle and dietary factors [full model, RRs across categories: 1.00, 0.97, 0.99, 0.96, 0.99, and 1.01; p-trend 0.46]. In contrast, yogurt consumption was associated with a significant lower risk of frailty in the age-adjusted model and after adjustment for lifestyle factors [RRs: 1.00, 0.95, 0.89, 0.89, and 0.97; p-trend < 0.001]. However, after additional adjustment for diet quality, the estimates increased and the association lost significance (RRs: 1.00, 1.00, 0.98, 1.01, and 1.14; p-trend 0.33). Women with a higher intake of cheese had a significant higher risk of frailty compared to those with a lower intake in all models (full model, RRs: 1.00, 1.04, 1.08, 1.11, and 1.17; p-trend < 0.001). Consumption was low for other dairy products including cream and ice cream, which were not associated with frailty (data now shown). Further adjustment for protein intake or physical activity did not change the results (Additional file 1: Supplemental Table 1). Cheese intake was positively associated with three of the five frailty criteria; low strength (RR per 1 serving/d increment: 1.11 (1.03, 1.19), reduced aerobic capacity (1.08; 1.00, 1.17), and the weight loss criterion (1.09; 1.00, 1.19) (Additional file 1: Supplemental Table 2). Stratification by BMI level shows that the association between cheese and frailty loses significance among those who have a BMI below 25 kg/m^2^ (Additional file 1: Supplemental Table 3). Also excluding participants with missing values on any of the frailty criteria attenuated the association between cheese consumption and frailty incidence, likely due to reduced power (data not shown).

**Table 2:** Relative risks (95% confidence interval) of frailty according to categories of dairy consumption among 85,280 women aged ≥ 60y in the Nurses’ Health Study

**Milk**	**Dairy categories**						***P* value**	**Per serving/d increment**
	** < 1/wk**	**1/wk**	**2 to 4 /wk**	**5 to 6 /wk**	**1/d**	** ≥ 2/d**		
Participants, n	11,584	8403	24,302	12,555	20,429	8007		
Person-yr	166,882	141,165	419,068	208,781	328,540	108,879		
Frailty cases, n	1734	1592	5029	2464	3935	1158		
Age-adjusted	1.00	1.00 (0.93, 1.07)	1.03 (0.98, 1.09)	1.01 (0.95, 1.08)	1.06 (1.00, 1.12)	1.04 (0.96, 1.12)	0.08	1.02 (1.00, 1.05)
Multivariable model^a^	1.00	0.96 (0.89, 1.02)	0.97 (0.92, 1.02)	0.94 (0.88, 1.00)	0.97 (0.91, 1.02)	0.98 (0.91, 1.06)	0.90	1.00 (0.98, 1.03)
Multivariable model^b^	1.00	0.97 (0.90, 1.03)	0.99 (0.93, 1.04)	0.96 (0.90, 1.02)	0.99 (0.94, 1.05)	1.01 (0.93, 1.09)	0.46	1.01 (0.99, 1.04)
**Yogurt**	**Never**	** < 1/wk**	**1/wk**	**2 to 4 /wk**	** ≥ 5/wk**			
Participants, n	24,495	33,1159	15,387	10,986	1253			
Person-yr	307,484	581,896	273,943	192,121	17,870			
Frailty cases, n	3168	7404	3148	2023	169			
Age-adjusted	1.00	0.96 (0.92, 1.01)	0.88 (0.84, 0.92)	0.83 (0.78, 0.87)	0.84 (0.72, 0.98)		< 0.001	0.67 (0.61, 0.74)
Multivariable model^a^	1.00	0.95 (0.91, 0.99)	0.89 (0.85, 0.94)	0.89 (0.84, 0.94)	0.97 (0.83, 1.14)		< 0.001	0.83 (0.75, 0.92)
Multivariable model^b^	1.00	1.00 (0.95, 1.04)	0.98 (0.93, 1.03)	1.01 (0.95, 1.07)	1.14 (0.97, 1.33)		0.33	1.06 (0.95, 1.18)
**Cheese**	** < 1/wk**	**1/wk**	**2 to 4 /wk**	**5 to 6 /wk**	** ≥ 1/d**			
Participants, n	4922	11,799	43,517	14,819	10,223			
Person-yr	76,670	200,193	731,455	226,683	138,313			
Frailty cases, n	794	2285	8654	2617	1562			
Age-adjusted	1.00	1.08 (0.99, 1.17)	1.14 (1.06, 1.22)	1.17 (1.08, 1.26)	1.24 (1.13, 1.35)		< 0.001	1.13 (1.08, 1.18)
Multivariable model^a^	1.00	1.04 (0.96, 1.13)	1.07 (0.99, 1.15)	1.08 (0.99, 1.17)	1.14 (1.04, 1.25)		0.003	1.08 (1.03, 1.13)
Multivariable model^b^	1.00	1.04 (0.96, 1.13)	1.08 (1.00, 1.17)	1.11 (1.02, 1.21)	1.17 (1.07, 1.28)		< 0.001	1.10 (1.05, 1.16)

^a^Cox regression model adjusted for: age (months), calendar time (4-y intervals), census tract income (< $45,000, $45,000–$59,999, $60,000–$74,999, $75,000–$99,999, or ≥ $100,000/y), education (registered nursing degrees, bachelor’s degree, masters or doctorate degree), baseline body mass index (< 25.0, 25.0–29.9, ≥ 30.0 kg/m^2^), smoking status (never, past, and current 1–14, 15–24, and ≥ 25 cigarettes/day), alcohol intake (0, 1.0–4.9, 5.0–14.9, or ≥ 15.0 g/d), energy intake (quintiles of kcal/d) and medication use (aspirin, postmenopausal hormone therapy, diuretics, β-blockers, calcium channel blockers, ACE inhibitors, other blood pressure medication, lipid lowering medication, insulin, and oral hypoglycemic medication)

^b^Adjustment as in the previous model and additionally adjusted for adherence to the Alternate Healthy Eating Index (quartiles). Milk, yogurt, and cheese were mutually adjusted for each other (all in categories)

The intake of low-fat milk did not result in a different effect on the risk of frailty when compared to whole milk [full model, per serving/d increase: low-fat milk 1.01 (0.99, 1.04), whole milk 1.01 (0.93, 1.09)] (Table 3). Also, plain yogurt did not show a significant different effect on frailty compared to sweetened yogurt after adjustment for diet quality [full model, per s/d increase: plain yogurt 1.01 (0.92, 1.10), sweetened yogurt 1.04 (0.91, 1.20)]. Higher consumption of low-fat cheese was significantly associated with the risk of frailty, while an increased high-fat cheese consumption was not significantly associated with risk of frailty [full model, per serving/d increase: low-fat cheese 1.26 (1.15, 1.37), high-fat cheese 1.04 (0.98, 1.10)].

**Table 3:** Relative risks (95% confidence interval) of frailty according to subcategories of milk, yogurt, and cheese among 85,280 women aged ≥ 60y in the Nurses’ Health Study

**Low-fat milk**	**Dairy categories**						***P* value**	**Per serving/d increment**
	** < 1/wk**	**1/wk**	**2 to 4 /wk**	**5 to 6 /wk**	**1/d**	** ≥ 2/d**		
Participants, n	18,024	9730	23,724	10,730	16,997	6075		
Person-yr	245,473	162,295	413,756	184,349	281,619	85,823		
Frailty cases, n	2467	1851	5023	2206	3438	927		
Age-adjusted	1.00	1.01 (0.95, 1.08)	1.02 (0.97, 1.07)	1.00 (0.95, 1.06)	1.04 (0.99, 1.10)	1.01 (0.94, 1.09)	0.34	1.02 (0.99, 1.04)
Multivariable model^a^	1.00	0.98 (0.92, 1.04)	0.96 (0.91, 1.01)	0.94 (0.88, 0.99)	0.95 (0.90, 1.00)	0.96 (0.89, 1.03)	0.16	0.99 (0.97, 1.02)
Multivariable model^b^	1.00	0.99 (0.93, 1.06)	0.99 (0.94, 1.04)	0.98 (0.93, 1.04)	1.00 (0.95, 1.06)	1.01 (0.93, 1.09)	0.70	1.01 (0.99, 1.04)
**Whole milk**	** < 1/wk**	**1/wk**	**2 to 4 /wk**	**5 to 6 /wk**	** ≥ 1/d**			
Participants, n	64,384	8316	8546	1683	2351			
Person-yr	1,081,896	132,340	116,060	20,374	22,645			
Frailty cases, n	12,834	1548	1160	185	185			
Age-adjusted	1.00	1.08 (1.02, 1.14)	1.01 (0.95, 1.08)	1.02 (0.88, 1.17)	1.17 (1.01, 1.35)		0.03	1.07 (0.99, 1.15)
Multivariable model^a^	1.00	1.08 (1.03, 1.14)	1.04 (0.98, 1.10)	1.07 (0.93, 1.24)	1.24 (1.07, 1.43)		< 0.001	1.12 (1.03, 1.20)
Multivariable model^b^	1.00	1.03 (0.98, 1.09)	0.97 (0.92, 1.04)	0.98 (0.85, 1.14)	1.15 (0.99, 1.33)		0.41	1.01 (0.93, 1.09)
**Plain yogurt**	**Never**	** < 1/wk**	**1/wk**	**2 to 4 /wk**	** ≥ 5/wk**			
Participants, n	45,147	19,278	4937	7839	2501			
Person-yr	610,104	460,222	97,819	134,140	30,734			
Frailty cases, n	6260	6601	1240	1506	242			
Age-adjusted	1.00	0.98 (0.94, 1.01)	0.90 (0.85, 0.96)	0.95 (0.90, 1.01)	0.90 (0.79, 1.02)		0.01	0.89 (0.81, 0.97)
Multivariable model^a^	1.00	0.95 (0.92, 0.99)	0.88 (0.83, 0.94)	0.92 (0.86, 0.97)	0.89 (0.78, 1.01)		< 0.001	0.85 (0.78, 0.93)
Multivariable model^b^	1.00	1.00 (0.96, 1.04)	0.96 (0.90, 1.03)	1.02 (0.96, 1.08)	1.01 (0.89, 1.15)		0.69	1.01 (0.92, 1.10)
**Sweetened yogurt**	**Never**	** < 1/wk**	**1/wk**	**2 to 4 /wk**	** ≥ 5/wk**			
Participants, n	50,587	20,012	3622	4472	1009			
Person-yr	696,624	484,291	67,644	73,079	11,381			
Frailty cases, n	7363	6916	843	650	77			
Age-adjusted	1.00	0.96 (0.93, 0.99)	0.90 (0.84, 0.97)	0.78 (0.72, 0.84)	0.85 (0.68, 1.07)		< 0.001	0.64 (0.55, 0.74)
Multivariable model^a^	1.00	0.99 (0.96, 1.03)	1.02 (0.95, 1.10)	0.94 (0.86, 1.02)	1.07 (0.86, 1.34)		0.50	0.96 (0.84, 1.10)
Multivariable model^b^	1.00	1.01 (0.98, 1.05)	1.06 (0.99, 1.14)	0.98 (0.90, 1.06)	1.13 (0.90, 1.42)		0.63	1.04 (0.91, 1.20)
**Low-fat cheese**	** < 1/wk**	**1/wk**	**2 to 4 /wk**	**5 to 6 /wk**	** ≥ 1/d**			
Participants, n	48,095	20,676	14,780	1173	556			
Person-yr	723,330	364,472	260,337	18,100	7075			
Frailty cases, n	7811	4428	3352	245	76			
Age-adjusted	1.00	1.03 (0.99, 1.07)	1.08 (1.03, 1.12)	1.18 (1.04, 1.34)	1.09 (0.87, 1.38)		< 0.001	1.17 (1.08, 1.28)
Multivariable model^a^	1.00	1.00 (0.96, 1.03)	1.02 (0.98, 1.06)	1.14 (1.00, 1.30)	1.12 (0.89, 1.40)		0.06	1.06 (0.97, 1.15)
Multivariable model^b^	1.00	1.05 (1.01, 1.09)	1.11 (1.06, 1.16)	1.26 (1.11, 1.44)	1.21 (0.96, 1.53)		< 0.001	1.26 (1.15, 1.37)
**High-fat cheese**	** < 1/wk**	**1/wk**	**2 to 4 /wk**	**5 to 6 /wk**	** ≥ 1/d**			
Participants, n	11,915	19,595	42,254	7219	4297			
Person-yr	218,197	359,160	648,107	94,930	52,920			
Frailty cases, n	2553	4472	7358	969	560			
Age-adjusted	1.00	1.11 (1.05, 1.16)	1.13 (1.08, 1.18)	1.12 (1.04, 1.21)	1.22 (1.11, 1.34)		< 0.001	1.12 (1.06, 1.18)
Multivariable model^a^	1.00	1.07 (1.02, 1.12)	1.08 (1.03, 1.13)	1.07 (0.99, 1.15)	1.19 (1.08, 1.31)		0.003	1.09 (1.03, 1.16)
Multivariable model^b^	1.00	1.05 (1.00, 1.10)	1.04 (0.99, 1.09)	1.01 (0.93, 1.09)	1.12 (1.02, 1.23)		0.18	1.04 (0.98, 1.10)

^a^Cox regression model adjusted for: age (months), calendar time (4-y intervals), census tract income (< $45,000, $45,000–$59,999, $60,000–$74,999, $75,000–$99,999, or ≥ $100,000/y), education (registered nursing degrees, bachelor’s degree, masters or doctorate degree), baseline body mass index (< 25.0, 25.0–29.9, ≥ 30.0 kg/m^2^), smoking status (never, past, and current 1–14, 15–24, and ≥ 25 cigarettes/day), alcohol intake (0, 1.0–4.9, 5.0–14.9, or ≥ 15.0 g/d), energy intake (quintiles of kcal/d) and medication use (aspirin, postmenopausal hormone therapy, diuretics, β-blockers, calcium channel blockers, ACE inhibitors, other blood pressure medication, lipid lowering medication, insulin, and oral hypoglycemic medication)

^b^Adjustment as in the previous model and additionally adjusted for adherence to the Alternate Healthy Eating Index (quartiles). Low-fat milk, whole milk, plain yogurt, sweetened yogurt, low-fat cheese, and high-fat cheese were mutually adjusted for each other (all in categories)

**Fig. 1:**
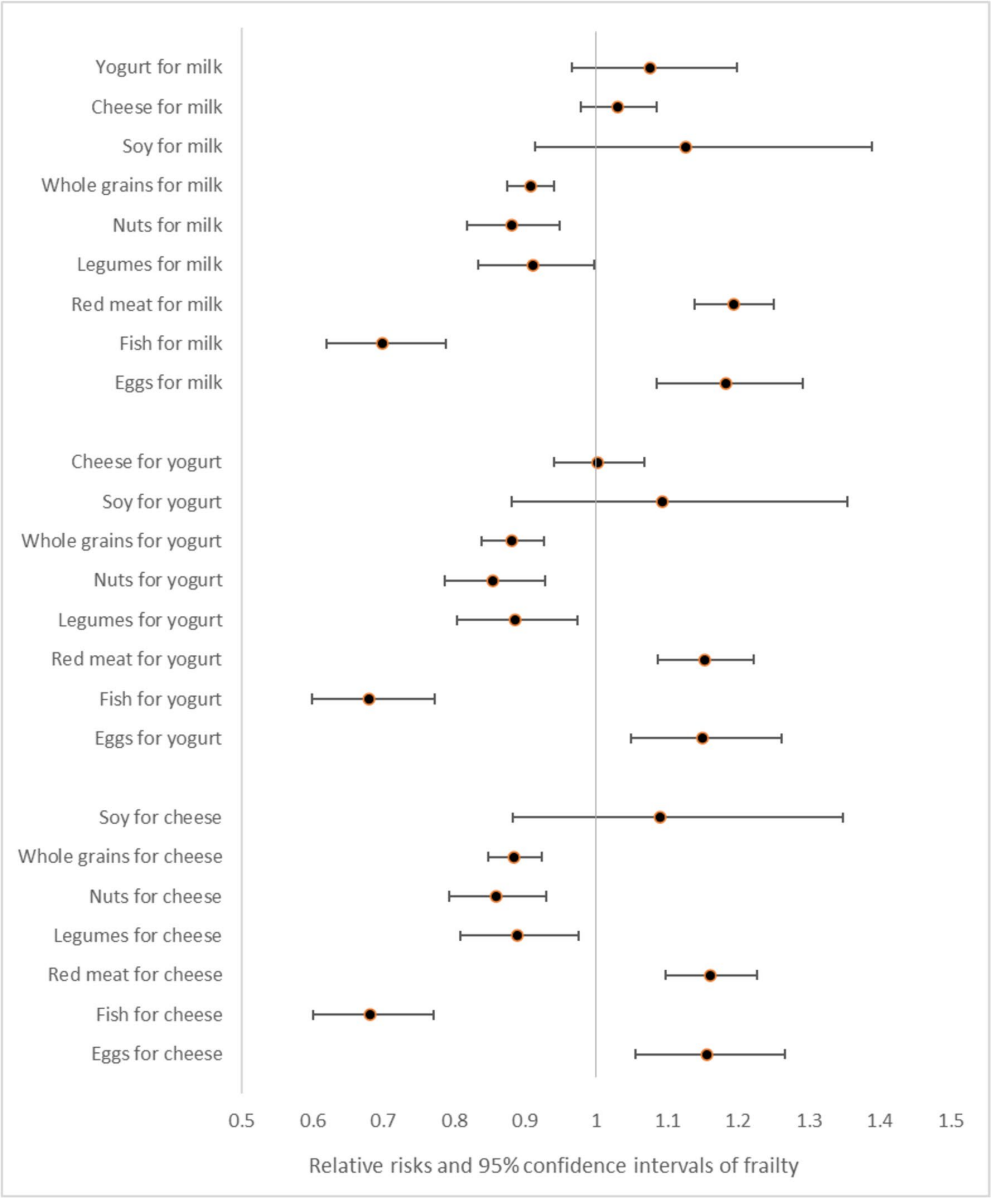
Relative risks (RR) and their 95% confidence interval of frailty for the replacement of 1 serving/d of milk, yogurt or cheese with different protein sources among women aged ≥ 60y in the Nurses’ Health Study. Multivariable model was adjusted for: age (months), calendar time (4-y intervals), census tract income (< $45,000, $45,000–$59,999, $60,000–$74,999, $75,000–$99,999, or ≥ $100,000/y), education (registered nursing degrees, bachelor’s degree, masters or doctorate degree), baseline body mass index (< 25.0, 25.0–29.9, ≥ 30.0 kg/m.^2^), smoking status (never, past, and current 1–14, 15–24, and ≥ 25 cigarettes/day), alcohol intake (0, 1.0–4.9, 5.0–14.9, or ≥ 15.0 g/d), energy intake (quintiles of kcal/d), medication use (aspirin, postmenopausal hormone therapy, diuretics, β-blockers, calcium channel blockers, ACE inhibitors, other blood pressure medication, lipid lowering medication, insulin, and oral hypoglycemic medication)

**Fig. 2:**
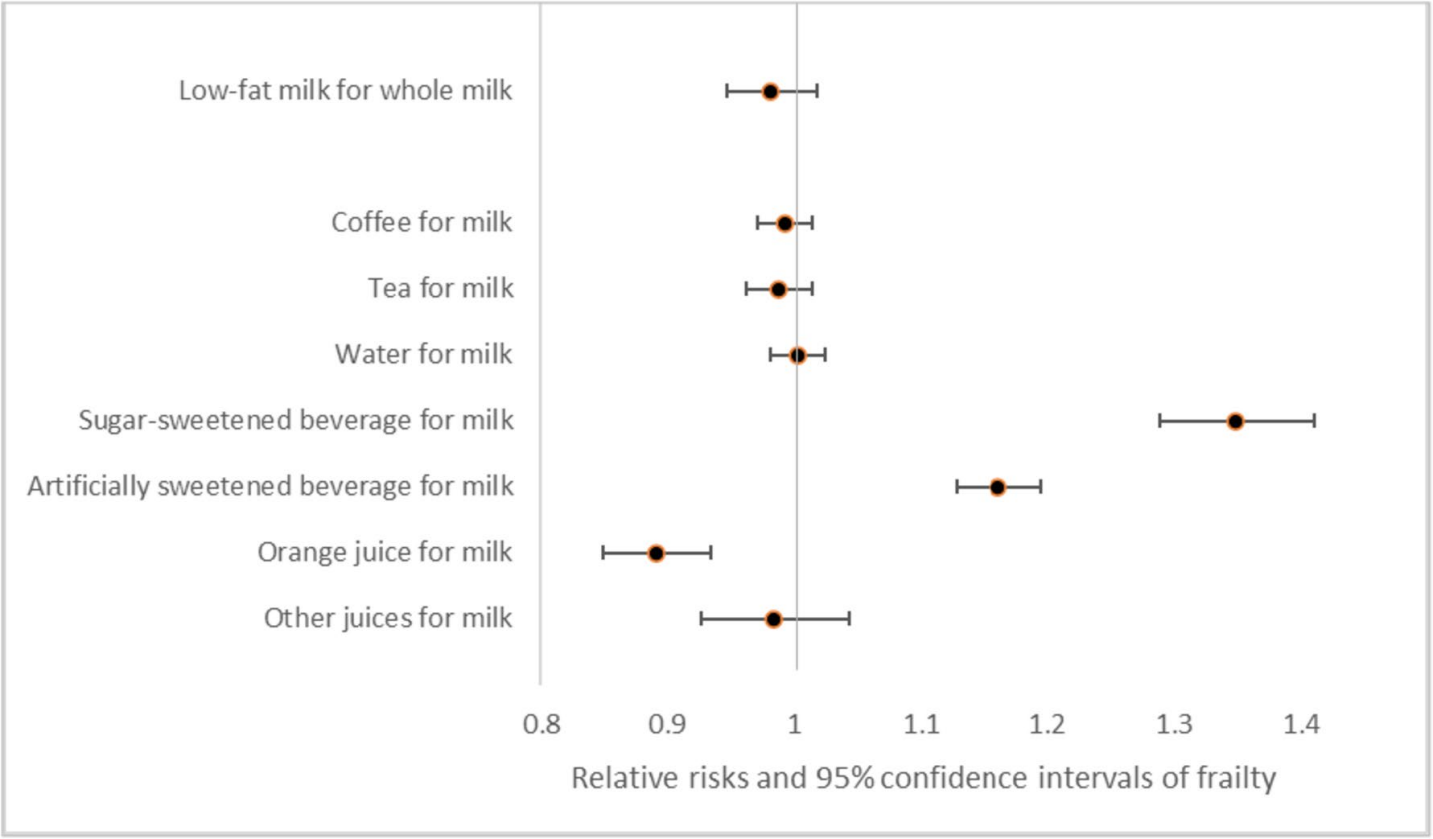
Relative risks (RR) and their 95% confidence interval of frailty for the replacement of 1 serving/d of milk with different beverages among women aged ≥ 60y in the Nurses’ Health Study. Multivariable model was adjusted for: age (months), calendar time (4-y intervals), census tract income (< $45,000, $45,000–$59,999, $60,000–$74,999, $75,000–$99,999, or ≥ $100,000/y), education (registered nursing degrees, bachelor’s degree, masters or doctorate degree), baseline body mass index (< 25.0, 25.0–29.9, ≥ 30.0 kg/m.^2^), smoking status (never, past, and current 1–14, 15–24, and ≥ 25 cigarettes/day), alcohol intake (0, 1.0–4.9, 5.0–14.9, or ≥ 15.0 g/d), energy intake (quintiles of kcal/d), medication use (aspirin, postmenopausal hormone therapy, diuretics, β-blockers, calcium channel blockers, ACE inhibitors, other blood pressure medication, lipid lowering medication, insulin, and oral hypoglycemic medication)

Replacing one serving a day of milk, yogurt, or cheese with an equal exchange of whole grains, nuts, legumes, or fish was associated with a decreased risk of frailty (Fig. 1). Replacing milk, yogurt, or cheese with red meat or eggs was associated with a significant increased risk of frailty. Additional adjustment for total protein intake or sugar sweetened beverages did not change the results of these substitution analysis (data not shown). When replacing milk with a sugar-sweetened beverage or artificially sweetened beverage an increased risk of frailty was seen (Fig. 2). On the contrary, replacing milk with orange juice resulted in a lower risk of frailty. The results did not change with additional adjustment for fruit and vegetables (data not shown).

Sensitivity analyses among prefrail women showed an attenuation of the detrimental association between cheese and frailty (Additional file 1: Supplemental Table 4). The magnitude of the associations remained similar when excluding persons with heart disease, diabetes, or cancer (Additional file 1: Supplemental Table 5). There was no difference in association between dairy and risk of frailty by latency period (Additional file 1: Supplemental Table 6).

## Discussion

In the present prospective analysis among 85,280 older adults, we found that women with a high cheese consumption had an increased risk of frailty. The consumption of low-fat milk, whole milk, plain or sweetened yogurt was not associated with the risk of frailty. When any type of dairy was replaced with whole grains, nuts, legumes, or fish the risk of frailty was lower; by contrast, when any type of dairy was replaced with red meat or eggs, the risk of frailty was higher. Furthermore, replacing milk with orange juice was associated with lower risk of frailty, while replacing milk with a sugar-sweetened beverage or artificially sweetened beverage was associated with higher risk of frailty.

In a Spanish study among 1871 older adults, with a follow-up of 3.5 years, compared to participants who consumed less than one serving of low-fat milk a week, those who consumed seven or more servings a week had a 43% lower risk of frailty as defined by the physical frailty phenotype; cheese consumption, as well as consumption of whole milk, whole yogurt or low-fat yogurt was not associated with frailty incidence [28]. Among 823 participants from the French Three-City Bordeaux study, no association was found between any dairy product, including milk, fresh dairy products (yogurt and ricotta cheese), and cheese and self-reported and performance-based definitions of frailty, after 10 years of follow-up [29]. In participants from the American Framingham Offspring Study (*n* = 2550) intakes of yogurt were modestly associated with reduced frailty onset and dietary intakes of high-fat dairy (whole milk, ice cream, cottage/ ricotta cheese and other cheese) had a borderline association with increased odds of frailty [30]. In contrast to the European studies, we found that consumption of cheese was associated with higher risk of frailty. Apart from the difference in study size, length of follow-up, and frailty definition, it is possible that the lifestyle and dietary patterns associated with the type of cheese consumption played a role. In the US, cheese is less often eaten alone and usually consumed as an ingredient of other foods and dishes, e.g., pizzas, burgers, or pastas. Therefore, it is possible that the association found between cheese and frailty might have captured the combined effect of these mixed dishes that represent a typical Western pattern, which is a known risk factor of frailty [31]. However, control for overall dietary quality did not alter our findings.

We have been unable to detect an inverse association of any type of dairy on frailty, despite the potentially beneficial nature of the dairy nutrients. In contrast to milk, yogurt showed a modest protective effect on frailty in our study after adjustment for medication use and several lifestyle factors but not after adjustment for overall diet quality. It is possible that this association did not reach significance due to relatively low intake levels of yogurt. Furthermore, in a population that already consumes adequate amounts of protein and other nutrients important for muscle health, it is possible that higher intake of dairy foods does not provide any substantial additional beneficial effect on preventing frailty.

The high saturated fat content of cheese might explain part of the detrimental association found between cheese and frailty and may also counterbalance the potential beneficial effects of nutrients in dairy such as calcium, magnesium, and vitamin D. High saturated fat intake increases LDL levels and may affect frailty because of its proinflammatory effect [32, 33]. This would suggest that dairy products lower in fat have a less detrimental effect compared to its high-fat equivalent. However, in our study we did not see a different association for low-fat milk as compared to whole milk. In addition, no attenuation of the results was found when only low-fat cheese was considered. It remains unclear why the association attenuated when assessing only high-fat cheese. This is in line with a recent 12-week randomized controlled trial among individuals with metabolic syndrome, which showed that consuming 3.3 servings of full-fat dairy/d in the form of milk, yogurt, and cheese does not significantly affect the fasting lipid profile or blood pressure compared to consuming identical amounts of low-fat dairy or a diet limited in dairy [34]. In another trial, a high daily intake of regular-fat cheese did not alter LDL cholesterol or metabolic syndrome risk factors differently than an equal intake of reduced-fat cheese [35]. In addition, several observational studies did not find a more favorable effect on health outcomes for low-fat compared to high-fat dairy products [36–38]. It has been suggested that dairy structures can enhance interactions in the dairy matrix that may modify the biological response to saturated fat from dairy [13],

Our substitution analysis provides evidence that the replacement food is of great importance for the effect of dairy on frailty. When dairy products are replaced with plant-based protein sources such as nuts, which provide high amounts of unsaturated fat, a lower risk is seen. In contrast, replacing dairy with red meat, which is also high in saturated fat, was associated with a higher risk of frailty. Similar findings were seen for the replacement of milk with other beverages. A glass of milk was associated with a lower risk compared to a sugar-sweetened beverage, while a glass of orange juice was associated with a lower risk compared to a glass of milk in relation to frailty. This suggests that the effect of dairy, regardless of its fat content, depends strongly on the food it replaces. These substitution analyses are meaningful since a decrease in one food leads to an increase in another food when the total energy intake remains stable. The use of serving sizes instead of grams has the advantage of being better interpretable for dietary recommendations. However, results of substitution analysis need to be interpreted cautiously since different foods may have been accompanied by a different meal. For example, milk is more likely to be consumed together with different foods compared to several other protein sources such as meat, which may result in residual confounding [39].

In addition to a person’s overall diet quality, overall health status might also be of importance in the relationship between dairy and frailty. The risk estimates for those with a yogurt consumption of ≥ 1 servings a day among prefrail women were more protective than those among robust women. However, the confidence intervals remained wide since the overall intake of yogurt was rather low in the cohort. In a Japanese study among 469 prefrail older adults, those who remained prefrail or even recovered after 2 years of follow-up had a significant higher milk and yogurt consumption compared to those that became frail [40]. Also, for older adults who are already malnourished or frail, dairy may be a palatable and digestible source of high-quality proteins. A recent RCT among institutionalized older adults, with deficiency in intake of calcium and protein intake, dietary supplementation for 2 years with dairy foods, including milk, yogurt, and cheese was associated with a 33% reduction in risk of fractures of any type, a 46% reduction in risk of hip fractures, and an 11% reduction in risk of falls, in comparison with the control group [41].

The strengths of the current study include a large sample size, and repeated assessments of dietary variables, covariates, and frailty over a very long follow-up. The current study is also subject to limitations. First, only one definition of frailty was used; our results should be confirmed in studies using other definitions of frailty that include performance-based measures such as the physical frailty phenotype [1]. Second, since dietary information was self-reported, measurement error and misclassification could occur. However, the FFQ used has been extensively validated against diet records and biomarkers and showed good correlations. Third, although we were able to adjust for many potential confounders including socioeconomic, lifestyle, clinical and dietary factors, residual and unmeasured confounding cannot be completely ruled out. Fourth, although studying the risk of frailty among only female nurses helped to increase internal validity, the observed associations might not apply to other populations. Lastly, reverse causation, although possible, seems unlikely because consistent results were found with long latencies between assessment of diet and incidence of frailty.

## Conclusions

Results from this study suggests that the effects of milk, yogurt, or cheese on the risk of frailty depend importantly on the replacing food products. Replacing any type of dairy with whole grains, nuts, legumes, or fish lowered the risk of frailty, while replacing any type of dairy with red meat or eggs, increased the risk of frailty. Furthermore, women with a high cheese consumption may have a modest increased risk of frailty, while consumption of milk and yogurt were not associated with risk of frailty.

### Supplementary Information

**Additional file 1: Supplemental Table 1.** Relative risks (95% confidence interval) of frailty according to categories of dairy consumption additionally adjusted for physical activity.; **Supplemental Table 2.** Relative risks (95% confidence interval) of frailty criteria per 1 serving/d increase of dairy consumption.; **Supplemental Table 3.** Relative risks (95% confidence interval) of frailty according to categories of dairy consumption among women with baseline BMI < 25 kg/m2 (n 43,307), 25—30 kg/m2 (n 25,560), and ≥ 30 kg/m2 (n 13,179).; **Supplemental Table 4.** Relative risks (95% confidence interval) of frailty according to categories of dairy consumption among women robust (n 45.374) or pre-frail (n 20,553) at baseline.; **Supplemental Table 5.** Relative risks (95% confidence interval) of frailty according to categories of dairy consumption among 71,580 women without cancer, diabetes, or heart disease.; **Supplemental Table 6.** Relative risks (95% confidence interval) of frailty according to categories of dairy consumption with varying latency periods.

## Data Availability

Information including the procedures to obtain and access data from the Nurses´ Health Studies is described at https://www.nurseshealthstudy.org/researchers.

## Abbreviations

AHEI: Alternate Healthy Eating Index
BMI: Body Mass Index
CI: Confidence Interval
FFQ: Food Frequency Questionnaire
NHS: Nurses´ Health Study
RDA: Recommended Dietary Allowance
RR: Relative Risk
SF-36: Short Form – 36
