# Supplementary material for: Specific dairy foods and risk of frailty in older women: a prospective cohort study
Source: BMC Med. 2024 Feb 29;22:89. doi: 10.1186/s12916-024-03280-8 (PMC10905813; doi:10.1186/s12916-024-03280-8)
Supplement: Supplementary file 1 — Additional file 1: Supplemental Table 1. Relative risks (95% confidence interval) of frailty according to categories of dairy consumption additionally adjusted for physical activity.; Supplemental Table 2. Relative risks (95% confidence interval) of frailty criteria per 1 serving/d increase of dairy consumption.; Supplemental Table 3. Relative risks (95% confidence interval) of frailty according to categories of dairy consumption among women with baseline BMI < 25 kg/m2 (n 43,307), 25—30 kg/m2 (n 25,560), and ≥ 30 kg/m2 (n 13,179).; Supplemental Table 4. Relative risks (95% confidence interval) of frailty according to categories of dairy consumption among women robust (n 45.374) or pre-frail (n 20,553) at baseline.; Supplemental Table 5. Relative risks (95% confidence interval) of frailty according to categories of dairy consumption among 71,580 women without cancer, diabetes, or heart disease.; Supplemental Table 6. Relative risks (95% confidence interval) of frailty according to categories of dairy consumption with varying latency periods. [file 12916_2024_3280_MOESM1_ESM.docx]

Supplemental material

**Specific dairy foods and risk of frailty in older women: a prospective cohort study**

Ellen A. Struijk, Teresa T. Fung, Fernando Rodriguez-Artalejo, Heike A. Bischoff-Ferrari,

Walter C. Willett^,^ Esther Lopez-Garcia.

Table of contents

**Supplemental table 1.** Relative risks (95% confidence interval) of frailty according to categories of dairy consumption additionally adjusted for physical activity.

**Supplemental table 2.** Relative risks (95% confidence interval) of frailty criteria per 1 serving/d increase of dairy consumption.

**Supplemental table 3**. Relative risks (95% confidence interval) of frailty according to categories of dairy consumption among women with baseline BMI <25 kg/m^2^ (n 43,307), 25 - 30 kg/m^2^ (n 25,560), and ≥30 kg/m^2^ (n 13,179).

**Supplemental table 4**. Relative risks (95% confidence interval) of frailty according to categories of dairy consumption among women robust (n 45.374) or pre-frail (n 20,553) at baseline.

**Supplemental table 5**. Relative risks (95% confidence interval) of frailty according to categories of dairy consumption among 71,580 women without cancer, diabetes, or heart disease.

**Supplemental table 6**. Relative risks (95% confidence interval) of frailty according to categories of dairy consumption with varying latency periods.

| **Supplemental table 1.** Relative risks (95% confidence interval) of frailty according to categories of dairy consumption additionally adjusted for physical activity or protein intake. | | | | | | | | | |
| --- | --- | --- | --- | --- | --- | --- | --- | --- | --- |
|  | **Dairy categories** | | | | | | **P value** | **Per serving/d increment** | |
| **Additional adjustment for physical activity** | | | |  |  |  |  |  | |
| **Milk** | **<1/wk** | **1/wk** | **2 to 4 /wk** | **5 to 6 /wk** | **1/d** | **≥2/d** |  |  | |
| Multivariable model^2^ | 1.00 | 0.97 (0.91, 1.04) | 1.00 (0.94, 1.06) | 0.98 (0.92, 1.04) | 1.01 (0.96, 1.08) | 1.04 (0.96, 1.12) | 0.13 | 1.02 (0.99, 1.05) |  |
| **Yogurt** | **Never** | **<1/wk** | **1/wk** | **2 to 4 /wk** | **≥5/wk** |  |  |  | |
| Multivariable model^2^ | 1.00 | 1.01 (0.96, 1.05) | 1.00 (0.95, 1.05) | 1.04 (0.98, 1.10) | 1.15 (0.98, 1.35) |  | 0.09 | 1.10 (0.99, 1.23) | |
| **Cheese** | **<1/wk** | **1/wk** | **2 to 4 /wk** | **5 to 6 /wk** | **≥1/d** |  |  |  | |
| Multivariable model^2^ | 1.00 | 1.05 (0.97, 1.14) | 1.10 (1.02, 1.18) | 1.13 (1.04, 1.23) | 1.20 (1.09, 1.31) |  | <0.001 | 1.12 (1.07, 1.17) | |
| **Additional adjustment for protein intake** | | | |  |  |  |  |  | |
| **Milk** | **<1/wk** | **1/wk** | **2 to 4 /wk** | **5 to 6 /wk** | **1/d** | **≥2/d** |  |  | |
| Multivariable model^2^ | 1.00 | 0.96 (0.90, 1.03) | 0.98 (0.93, 1.04) | 0.95 (0.89, 1.01) | 0.98 (0.92, 1.04) | 0.98 (0.91, 1.07) | 0.93 | 1.00 (0.98, 1.03) | |
| **Yogurt** | **Never** | **<1/wk** | **1/wk** | **2 to 4 /wk** | **≥5/wk** |  |  |  | |
| Multivariable model^2^ | 1.00 | 1.00 (0.95, 1.04) | 0.98 (0.93, 1.03) | 1.01 (0.95, 1.07) | 1.13 (0.96, 1.32) |  | 0.06 | 1.04 (0.94, 1.17) | |
| **Cheese** | **<1/wk** | **1/wk** | **2 to 4 /wk** | **5 to 6 /wk** | **≥1/d** |  |  |  | |
| Multivariable model^2^ | 1.00 | 1.04 (0.96, 1.13) | 1.07 (1.00, 1.16) | 1.10 (1.01, 1.19) | 1.15 (1.05, 1.26) |  | <0.001 | 1.12 (1.07, 1.17) | |
| ^1^Cox regression model adjusted for: age (months), calendar time (4-y intervals), census tract income (<$45,000, $45,000–$59,999, $60,000–$74,999, $75,000–$99,999, or ≥$100,000/y), education (registered nursing degrees, bachelor’s degree, masters or doctorate degree), baseline body mass index (<25.0, 25.0-29.9, ≥30.0 kg/m2), smoking status (never, past, and current 1-14, 15-24, and ≥25 cigarettes/day), alcohol intake (0, 1.0-4.9, 5.0-14.9, or ≥15.0 g/d), energy intake (quintiles of kcal/d) and medication use (aspirin, postmenopausal hormone therapy, diuretics, β-blockers, calcium channel blockers, ACE inhibitors, other blood pressure medication, lipid lowering medication, insulin, and oral hypoglycemic medication), adherence to the Alternate Healthy Eating Index (quartiles). Milk, yogurt, and cheese were mutually adjusted for each other (all in quintiles), and additionally adjusted for physical activity (quintiles of METs-h/wk), or protein intake (quintiles). | | | | | | | | | |

| **Supplemental table 2.** Relative risks (95% confidence interval) of frailty criteria per 1 serving/d increase of dairy consumption. | | | | | |
| --- | --- | --- | --- | --- | --- |
|  | **Fatigue** | **Low strength** | **Reduced aerobic capacity** | **≥5 chronic illnesses** | **Weight loss ≥5%** |
| **Milk** |  |  |  |  |  |
| Age-adjusted | 1.01 (0.97, 1.05) | 1.02 (0.98, 1.06) | 1.01 (0.97, 1.05) | 1.04 (0.98, 1.10) | 1.04 (1.00, 1.09) |
| Multivariable model^1^ | 1.01 (0.96, 1.05) | 1.02 (0.98, 1.06) | 1.01 (0.97, 1.06) | 0.97 (0.92, 1.04) | 1.01 (0.97, 1.06) |
| Multivariable model^2^ | 1.01 (0.97, 1.05) | 1.02 (0.99, 1.06) | 1.01 (0.97, 1.06) | 0.98 (0.92, 1.04) | 1.03 (0.98, 1.07) |
| **Yogurt** |  |  |  |  |  |
| Age-adjusted | 0.80 (0.68, 0.93) | 0.74 (0.64, 0.86) | 0.84 (0.71, 0.99) | 0.64 (0.50, 0.82) | 0.66 (0.55, 0.79) |
| Multivariable model^1^ | 0.99 (0.84, 1.17) | 0.95 (0.81, 1.10) | 0.98 (0.82, 1.16) | 0.71 (0.55, 0.92) | 0.79 (0.65, 0.95) |
| Multivariable model^2^ | 1.21 (1.03, 1.44) | 1.16 (0.99, 1.35) | 1.08 (0.91, 1.29) | 0.92 (0.71, 1.20) | 1.03 (0.85, 1.24) |
| **Cheese** |  |  |  |  |  |
| Age-adjusted | 1.05 (0.97, 1.13) | 1.10 (1.03, 1.17) | 1.09 (1.01, 1.17) | 1.12 (1.01, 1.25) | 1.09 (1.01, 1.18) |
| Multivariable model^1^ | 1.03 (0.95, 1.12) | 1.09 (1.02, 1.17) | 1.08 (0.99, 1.17) | 0.92 (0.81, 1.04) | 1.06 (0.97, 1.15) |
| Multivariable model^2^ | 1.05 (0.97, 1.13) | 1.11 (1.03, 1.19) | 1.08 (1.00, 1.17) | 0.94 (0.83, 1.06) | 1.09 (1.00, 1.19) |
| ^1^1Cox regression model adjusted for: age (months), calendar time (4-y intervals), census tract income (<$45,000, $45,000–$59,999, $60,000–$74,999, $75,000–$99,999, or ≥$100,000/y), education (registered nursing degrees, bachelor’s degree, masters or doctorate degree), baseline body mass index (<25.0, 25.0-29.9, ≥30.0 kg/m2), smoking status (never, past, and current 1-14, 15-24, and ≥25 cigarettes/day), alcohol intake (0, 1.0-4.9, 5.0-14.9, or ≥15.0 g/d), energy intake (quintiles of kcal/d) and medication use (aspirin, postmenopausal hormone therapy, diuretics, β-blockers, calcium channel blockers, ACE inhibitors, other blood pressure medication, lipid lowering medication, insulin, and oral hypoglycemic medication).  ^2^Adjustment as in the previous model and additionally adjusted for adherence to the Alternate Healthy Eating Index (quartiles). Milk, yogurt, and cheese were mutually adjusted for each other (all in quintiles). | | | | | |

| **Supplemental table 3**. Relative risks (95% confidence interval) of frailty according to categories of dairy consumption among women with baseline BMI <25 kg/m^2^ (n 43,307), 25 - 30 kg/m^2^ (n 25,560), and ≥30 kg/m^2^ (n 13,179). | | | | | | | | | |
| --- | --- | --- | --- | --- | --- | --- | --- | --- | --- |
|  | **Dairy categories** | | | | | | **P value** | **Per serving/d increment** | |
| **Milk** | **<1/wk** | **1/wk** | **2 to 4 /wk** | **5 to 6 /wk** | **1/d** | **≥2/d** |  |  | |
| BMI <25 kg/m^2^ | 1.00 | 0.91 (0.82, 1.02) | 0.96 (0.88, 1.05) | 0.87 (0.78, 0.97) | 0.92 (0.83, 1.01) | 1.03 (0.91, 1.17) | 0.80 | 1.01 (0.98, 1.06) |  |
| BMI 25 - 30 kg/m^2^ | 1.00 | 0.93 (0.83, 1.05) | 0.93 (0.84, 1.03) | 0.97 (0.87, 1.08) | 0.99 (0.89, 1.10) | 0.92 (0.80, 1.06) | 0.88 | 1.01 (0.96, 1.05) |  |
| BMI ≥30 kg/m^2^ | 1.00 | 1.11 (0.96, 1.28) | 1.07 (0.95, 1.21) | 1.07 (0.94, 1.21) | 1.09 (0.96, 1.23) | 1.05 (0.89, 1.23 | 0.82 | 1.01 (0.96, 1.06) |  |
| **Yogurt** | **Never** | **<1/wk** | **1/wk** | **2 to 4 /wk** | **≥5/wk** |  |  |  | |
| BMI <25 kg/m^2^ | 1.00 | 0.94 (0.88, 1.02) | 0.93 (0.86, 1.02) | 0.93 (0.84, 1.02) | 1.13 (0.89, 1.44) |  | 0.82 | 1.00 (0.84, 1.20) | |
| BMI 25 - 30 kg/m^2^ | 1.00 | 1.01 (0.94, 1.09) | 1.00 (0.91, 1.10) | 1.05 (0.95, 1.17) | 1.14 (0.85, 1.54) |  | 0.29 | 1.13 (0.93, 1.35) | |
| BMI ≥30 kg/m^2^ | 1.00 | 1.06 (0.97, 1.15) | 1.01 (0.91, 1.12) | 1.13 (1.00, 1.27) | 1.11 (0.80, 1.53) |  | 0.26 | 1.12 (0.90, 1.39) | |
| **Cheese** | **<1/wk** | **1/wk** | **2 to 4 /wk** | **5 to 6 /wk** | **≥1/d** |  |  |  | |
| BMI <25 kg/m^2^ | 1.00 | 1.04 (0.91, 1.18) | 1.11 (0.98, 1.24) | 1.03 (0.90, 1.18) | 1.16 (1.00, 1.34) |  | 0.17 | 1.06 (0.97, 1.15) | |
| BMI 25 - 30 kg/m^2^ | 1.00 | 1.09 (0.94, 1.26) | 1.12 (0.99, 1.28) | 1.14 (0.99, 1.32) | 1.19 (1.02, 1.39) |  | 0.05 | 1.08 (0.99, 1.18) | |
| BMI ≥30 kg/m^2^ | 1.00 | 1.05 (0.88, 1.26) | 1.07 (0.90, 1.26) | 1.24 (1.04, 1.49) | 1.23 (1.02, 1.49) |  | <0.001 | 1.21 (1.10, 1.33) | |
| ^*^We identified 5610 incident frailty cases among women with baseline BMI <25, 5513 incident frailty cases among women with baseline BMI 25-30 kg/m^2^, and 4290 incident frailty cases among women with BMI ≥30 kg/m^2^.  ^1^Cox regression model adjusted for: age (months), calendar time (4-y intervals), census tract income (<$45,000, $45,000–$59,999, $60,000–$74,999, $75,000–$99,999, or ≥$100,000/y), education (registered nursing degrees, bachelor’s degree, masters or doctorate degree), smoking status (never, past, and current 1-14, 15-24, and ≥25 cigarettes/day), alcohol intake (0, 1.0-4.9, 5.0-14.9, or ≥15.0 g/d), energy intake (quintiles of kcal/d), medication use (aspirin, postmenopausal hormone therapy, diuretics, β-blockers, calcium channel blockers, ACE inhibitors, other blood pressure medication, lipid lowering medication, insulin, and oral hypoglycemic medication), adherence to the Alternate Healthy Eating Index (quartiles). Milk, yogurt, and cheese were mutually adjusted for each other (all in quintiles). | | | | | | | | | |

| **Supplemental table 4**. Relative risks (95% confidence interval) of frailty according to categories of dairy consumption among women robust (n 45.374) or pre-frail (n 20,553) at baseline. | | | | | | | | | |
| --- | --- | --- | --- | --- | --- | --- | --- | --- | --- |
|  | **Dairy categories** | | | | | | **P value** | **Per serving/d increment** | |
| **Milk** | **<1/wk** | **1/wk** | **2 to 4 /wk** | **5 to 6 /wk** | **1/d** | **≥2/d** |  |  | |
| Robust (0 frailty criteria) | |  |  |  |  |  |  |  |  |
| Multivariable model^2^ | 1.00 | 0.98 (0.88, 1.09) | 0.98 (0.90, 1.07) | 0.98 (0.89, 1.07) | 0.99 (0.90, 1.08) | 1.00 (0.89, 1.13) | 0.84 | 1.01 (0.97, 1.05) |  |
| Prefrail (1 or 2 frailty criteria) |  |  |  |  |  |  |  |  |  |
| Multivariable model^1^ | 1.00 | 0.96 (0.86, 1.08) | 1.02 (0.93, 1.12) | 0.97 (0.87, 1.08) | 1.01 (0.91, 1.11) | 1.03 (0.91, 1.18) | 0.60 | 1.01 (0.97, 1.06) |  |
| **Yogurt** | **Never** | **<1/wk** | **1/wk** | **2 to 4 /wk** | **≥5/wk** |  |  |  | |
| Robust (0 frailty criteria) | |  |  |  |  |  |  |  | |
| Multivariable model^1^ | 1.00 | 0.98 (0.92, 1.05) | 0.99 (0.92, 1.08) | 1.04 (0.95, 1.14) | 1.06 (0.83, 1.35) |  | 0.16 | 1.11 (0.95, 1.31) | |
| Prefrail (1 or 2 frailty criteria) | |  |  |  |  |  |  |  | |
| Multivariable model^1^ | 1.00 | 0.98 (0.91, 1.05) | 0.89 (0.81, 0.97) | 0.98 (0.88, 1.08) | 0.93 (0.70, 1.23) |  | 0.27 | 0.91 (0.75, 1.10) | |
| **Cheese** | **<1/wk** | **1/wk** | **2 to 4 /wk** | **5 to 6 /wk** | **≥1/d** |  |  |  | |
| Robust (0 frailty criteria) | |  |  |  |  |  |  |  | |
| Multivariable model^1^ | 1.00 | 1.13 (0.99, 1.28) | 1.15 (1.02, 1.30) | 1.17 (1.03, 1.33) | 1.21 (1.05, 1.39) |  | 0.04 | 1.07 (0.99, 1.15) | |
| Prefrail (1 or 2 frailty criteria) | |  |  |  |  |  |  |  | |
| Multivariable model^1^ | 1.00 | 0.92 (0.80, 1.06) | 0.99 (0.87, 1.12) | 1.01 (0.88, 1.17) | 1.03 (0.89, 1.21) |  | 0.09 | 1.07 (0.99, 1.17) | |
| ^*^In the robust sample we identified 7143 incident frailty cases and in the pre-frail sample, we identified 5638 incident frailty cases.  ^1^Cox regression model adjusted for: age (months), calendar time (4-y intervals), census tract income (<$45,000, $45,000–$59,999, $60,000–$74,999, $75,000–$99,999, or ≥$100,000/y), education (registered nursing degrees, bachelor’s degree, masters or doctorate degree), baseline body mass index (<25.0, 25.0-29.9, ≥30.0 kg/m2), smoking status (never, past, and current 1-14, 15-24, and ≥25 cigarettes/day), alcohol intake (0, 1.0-4.9, 5.0-14.9, or ≥15.0 g/d), energy intake (quintiles of kcal/d), medication use (aspirin, postmenopausal hormone therapy, diuretics, β-blockers, calcium channel blockers, ACE inhibitors, other blood pressure medication, lipid lowering medication, insulin, and oral hypoglycemic medication), adherence to the Alternate Healthy Eating Index (quartiles). Milk, yogurt, and cheese were mutually adjusted for each other (all in quintiles). | | | | | | | | | |

| **Supplemental table 5**. Relative risks (95% confidence interval) of frailty according to categories of dairy consumption among 71,580 women without cancer, diabetes, or heart disease. | | | | | | | | | |
| --- | --- | --- | --- | --- | --- | --- | --- | --- | --- |
|  | **Dairy categories** | | | | | | **P value** | **Per serving/d increment** | |
| **Milk** | **<1/wk** | **1/wk** | **2 to 4 /wk** | **5 to 6 /wk** | **1/d** | **≥2/d** |  |  | |
| Multivariable model^2^ | 1.00 | 0.97 (0.88, 1.06) | 0.97 (0.90, 1.05) | 0.94 (0.86, 1.02) | 0.98 (0.91, 1.07) | 1.00 (0.90, 1.11) | 0.71 | 1.01 (0.98, 1.05) |  |
| **Yogurt** | **Never** | **<1/wk** | **1/wk** | **2 to 4 /wk** | **≥5/wk** |  |  |  | |
| Multivariable model^2^ | 1.00 | 1.00 (0.94, 1.06) | 0.98 (0.91, 1.05) | 1.02 (0.94, 1.11) | 1.13 (0.91, 1.39) |  | 0.36 | 1.06 (0.92, 1.22) | |
| **Cheese** | **<1/wk** | **1/wk** | **2 to 4 /wk** | **5 to 6 /wk** | **≥1/d** |  |  |  | |
| Multivariable model^2^ | 1.00 | 1.13 (1.00, 1.26) | 1.16 (1.05, 1.29) | 1.20 (1.07, 1.35) | 1.30 (1.14, 1.47) |  | <0.001 | 1.14 (1.06, 1.21) | |
| ^*^We identified 8343 incident frailty cases among women without cancer, diabetes, or heart disease.  ^1^Cox regression model adjusted for: age (months), calendar time (4-y intervals), census tract income (<$45,000, $45,000–$59,999, $60,000–$74,999, $75,000–$99,999, or ≥$100,000/y), education (registered nursing degrees, bachelor’s degree, masters or doctorate degree), baseline body mass index (<25.0, 25.0-29.9, ≥30.0 kg/m2), smoking status (never, past, and current 1-14, 15-24, and ≥25 cigarettes/day), alcohol intake (0, 1.0-4.9, 5.0-14.9, or ≥15.0 g/d), energy intake (quintiles of kcal/d) and medication use (aspirin, postmenopausal hormone therapy, diuretics, β-blockers, calcium channel blockers, ACE inhibitors, other blood pressure medication, lipid lowering medication, insulin, and oral hypoglycemic medication), adherence to the Alternate Healthy Eating Index (quartiles). Milk, yogurt, and cheese were mutually adjusted for each other (all in quintiles). | | | | | | | | | |

| **Supplemental table 6**. Relative risks (95% confidence interval) of frailty according to categories of dairy consumption with varying latency periods. | | | | | | | | | |
| --- | --- | --- | --- | --- | --- | --- | --- | --- | --- |
|  | **Dairy categories** | | | | | | **P value** | **Per serving/d increment** | |
| **Milk** | **<1/wk** | **1/wk** | **2 to 4 /wk** | **5 to 6 /wk** | **1/d** | **≥2/d** |  |  | |
| 0-6 years lag | 1.00 | 1.00 (0.93, 1.07) | 0.91 (0.86, 0.96) | 0.94 (0.88, 1.00) | 0.92 (0.88, 0.97) | 0.95 (0.90, 1.01) | 0.14 | 0.99 (0.97, 1.01) |  |
| 6-10 years lag | 1.00 | 0.98 (0.92, 1.06) | 1.00 (0.94, 1.06) | 0.96 (0.90, 1.02) | 1.00 (0.94, 1.07) | 1.01 (0.93, 1.09) | 0.64 | 1.01 (0.98, 1.03) |  |
| 10-14 years lag | 1.00 | 1.02 (0.95, 1.10) | 0.99 (0.93, 1.05) | 0.99 (0.92, 1.05) | 0.97 (0.91, 1.03) | 1.00 (0.92, 1.09) | 0.54 | 0.99 (0.97, 1.02) |  |
| **Yogurt** | **Never** | **<1/wk** | **1/wk** | **2 to 4 /wk** | **≥5/wk** |  |  |  | |
| 0-6 years lag | 1.00 | 0.95 (0.90, 0.99) | 0.98 (0.93, 1.03) | 0.98 (0.93, 1.03) | 1.03 (0.95, 1.13) |  | 0.61 | 1.04 (0.98, 1.11) | |
| 6-10 years lag | 1.00 | 0.98 (0.94, 1.02) | 0.97 (0.92, 1.02) | 0.98 (0.92, 1.04) | 1.02 (0.87, 1.20) |  | 0.77 | 0.99 (0.88, 1.10) | |
| 10-14 years lag | 1.00 | 0.99 (0.94, 1.03) | 0.97 (0.91, 1.02) | 0.95 (0.89, 1.02) | 1.01 (0.85, 1.19) |  | 0.25 | 0.94 (0.84, 1.06) | |
| **Cheese** | **<1/wk** | **1/wk** | **2 to 4 /wk** | **5 to 6 /wk** | **≥1/d** |  |  |  | |
| 0-6 years lag | 1.00 | 1.03 (0.97, 1.09) | 1.02 (0.97, 1.07) | 1.08 (1.01, 1.15) | 1.07 (1.00, 1.14) |  | 0.02 | 1.05 (1.01, 1.08) | |
| 6-10 years lag | 1.00 | 1.06 (0.97, 1.15) | 1.10 (1.01, 1.18) | 1.11 (1.02, 1.21) | 1.16 (1.06, 1.28) |  | 0.001 | 1.09 (1.04, 1.15) | |
| 10-14 years lag | 1.00 | 1.02 (0.93, 1.11) | 1.06 (0.97, 1.15) | 1.09 (0.99, 1.19) | 1.10 (1.00, 1.21) |  | 0.01 | 1.08 (1.02, 1.13) | |
| ^1^Cox regression model adjusted for: age (months), calendar time (4-y intervals), census tract income (<$45,000, $45,000–$59,999, $60,000–$74,999, $75,000–$99,999, or ≥$100,000/y), education (registered nursing degrees, bachelor’s degree, masters or doctorate degree), baseline body mass index (<25.0, 25.0-29.9, ≥30.0 kg/m2), smoking status (never, past, and current 1-14, 15-24, and ≥25 cigarettes/day), alcohol intake (0, 1.0-4.9, 5.0-14.9, or ≥15.0 g/d), energy intake (quintiles of kcal/d) and medication use (aspirin, postmenopausal hormone therapy, diuretics, β-blockers, calcium channel blockers, ACE inhibitors, other blood pressure medication, lipid lowering medication, insulin, and oral hypoglycemic medication), adherence to the Alternate Healthy Eating Index (quartiles). Milk, yogurt, and cheese were mutually adjusted for each other (all in quintiles). | | | | | | | | | |
